# The habenula: an under-recognised area of importance in frontotemporal dementia?

**DOI:** 10.1136/jnnp-2015-312067

**Published:** 2015-11-13

**Authors:** Martina Bocchetta, Elizabeth Gordon, Charles R Marshall, Catherine F Slattery, M Jorge Cardoso, David M Cash, Miklos Espak, Marc Modat, Sebastien Ourselin, Giovanni B Frisoni, Jonathan M Schott, Jason D Warren, Jonathan D Rohrer

**Affiliations:** 1 Department of Neurodegenerative Disease, Dementia Research Centre, UCL Institute of Neurology, London, UK; 2 Laboratory of Alzheimer's Neuroimaging and Epidemiology, IRCCS Istituto Centro San Giovanni di Dio—Fatebenefratelli, Brescia, Italy; 3 Department of Molecular and Translational Medicine, University of Brescia, Brescia, Italy; 4 Translational Imaging Group, Centre for Medical Image Computing (CMIC), University College London, London, UK; 5 Memory Clinic and Laboratory of Neuroimaging of Aging, University Hospitals and University of Geneva, Geneva, Switzerland

**Keywords:** DEMENTIA, MRI, NEUROANATOMY

## Introduction

Behavioural variant frontotemporal dementia (bvFTD) is a neurodegenerative disorder characterised by atrophy of the frontal and temporal lobes and progressive behavioural and cognitive impairment. Some behavioural symptoms such as craving for food, alcohol or drugs, and hypersexuality are suggestive of abnormal reward processing. The reward circuit is formed by a number of different structures including the orbitofrontal cortex, ventral striatum (in particular the nucleus accumbens), ventral pallidum, anterior cingulate cortex, thalamus, hypothalamus, midbrain and habenula.^1^ This complex network combines information about motivation, cognitive planning and motor control to develop an appropriate goal-directed response to external environmental stimuli. Many of the brain structures belonging to the reward circuit have been found to be atrophic in bvFTD,^2^ supporting the theory that impairment of the reward system is an important factor in this disease. Among these structures, the habenula, found medial to the posterior thalamus, is uniquely positioned to participate in reward processing, acting as a convergence point for the limbic system and basal ganglia circuits,^3^ ^4^ and therefore playing a pivotal role in the integration of information required to generate goal-directed behaviours. Despite this key role, it has yet to be investigated in bvFTD.

The aim of this study was to investigate the volume of the habenula in a cohort of patients with bvFTD, hypothesising that it would be smaller than in healthy controls as well as an age-matched group of patients with Alzheimer’s disease (AD) who typically do not show impairment of reward behaviour. We also hypothesised that the habenula would show comparable atrophy to other key areas in the reward network in bvFTD.

## Methods

Fifteen participants fulfilling criteria for the diagnosis of bvFTD (including eight with a *MAPT* mutation and four with a pathogenic expansion in the *C9orf72* gene) were recruited consecutively from a tertiary referral cognitive disorders clinic at the National Hospital for Neurology and Neurosurgery, London, UK. In total, 87% of the group were male with the mean (SD) age at onset 55.3 (8.9) years and disease duration 7.3 (3.8) years. Fifteen participants fulfilling criteria for typical AD (with early onset disease in order to match for age) were also recruited. Only 40% of the group were male with the mean (SD) age at onset 54.9 (4.5) years and disease duration 5.9 (2.7) years. Fifteen healthy controls (47% male) were also recruited. The mean (SD) age at scan was 62.6 (9.8) in bvFTD, 60.7 (5.9) in AD and 61.4 (8.9) in the controls, with no significant differences between the groups. Mini-Mental State Examination differed between the groups, being lowest in the AD group (20.4 (4.2)) then the bvFTD group (25.0 (4.6)) (AD vs bvFTD, p=0.011), both being lower than the control group (28.9 (1.3), p<0.001 and 0.055, respectively).

Segmentations of the habenula were performed manually on coronal slices of a volumetric T1-weighted MRI following a novel segmentation protocol adapted from previous descriptions^5^ ^6^ (see online supplementary data). We also calculated volumes for the rest of the brain using a cortical and subcortical parcellation as previously described,^7^ (see online supplementary data). All brain volumes were corrected for total intracranial volume, which was calculated using SPM12 (http://www.fil.ion.ucl.ac.uk/spm).

Statistical analyses were performed in SPSS software V.22.0 (SPSS Inc, Chicago, Illinois, USA). Differences in demographic and cognitive features as well as brain volumes were tested with the Mann–Whitney U test for continuous variables and χ^2^ test for dichotomous variables. For the brain volumes (30 comparisons), the Bonferroni correction for multiple comparisons was made so that only a threshold of p≤0.001 was considered significant.

## Results

**Figure 1:**
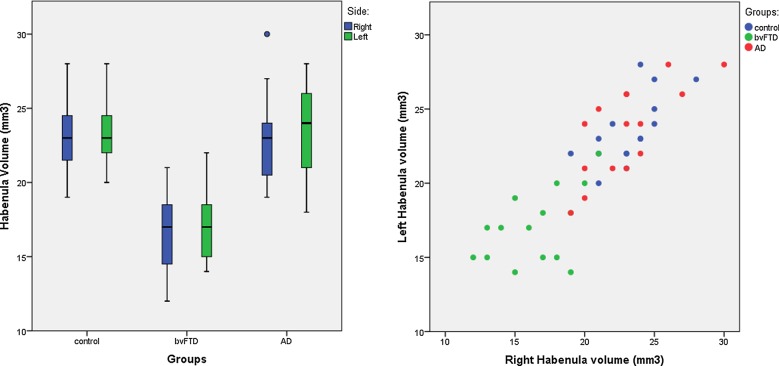
Volume of the left and right habenula (corrected for total intracranial volume) in 15 patients with behavioural variant frontotemporal dementia (bvFTD), 15 patients with Alzheimer's disease (AD) and 15 controls: (A) by group and (B) comparing the right and left side.

The bvFTD group showed a 30% lower right and a 28% lower left habenular volume compared with controls (mean (SD) right: 16.4 (2.7) vs 23.3 (2.2) mm^3^, left: 16.9 (2.4) vs 23.6 (2.2), p<0.0005, Mann-Whitney U test). The AD group was not significantly different to controls (<1% difference): mean (SD) right: 23.0 (2.9), left: 23.6 (3.1), but the bvFTD group was significantly smaller than AD (right 29% and left 28% smaller, p<0.0005 for both sides) (figure 1).

No other cortical or subcortical region showed a larger percentage difference in volume in bvFTD compared with controls than the habenula (see online supplementary table). The insula cortex, amygdala, hippocampus and nucleus accumbens were the other most significantly involved regions, with volumes being 20% or smaller than controls. Other areas of the reward network including the frontal and cingulate cortices, and thalamus showed smaller volumetric differences compared with controls (see online supplementary table).

## Discussion

To the best of our knowledge, this is the first study investigating the habenula in bvFTD. Compared with healthy controls and patients with AD, bvFTD showed significantly smaller habenular volumes bilaterally. Furthermore, the habenula showed the largest percentage difference in volume in the bvFTD group compared with controls out of all of the cortical and subcortical regions measured. Similarly affected regions included the nucleus accumbens, amygdala, hippocampus and insula cortex, which form part of the reward network or are intrinsically linked to it. Other key areas of the reward network including the thalamus and brainstem were affected to a lesser extent. However, the key areas within the network form smaller parts of the regions measured in this study (ventral part of the pallidum, dorsomedial nucleus of the thalamus and midbrain) and it may be that subsegmentation of these regions would show more specific involvement in these particular subregions.

The habenula is involved in the processing of aversive information. By inhibiting dopamine-releasing neurones, it suppresses motor activity under adverse conditions such as failure to obtain a reward or anticipation of an unpleasant outcome.^8^ For example, in a motion-prediction fMRI task the habenula was activated when a subject received feedback indicating that their response was wrong.^9^ When the action of the habenula is impaired (such as when it becomes atrophied), it is likely that even though the outcome of an action may be negative, it would be difficult for a subject to avoid the action. This may be expressed as abnormal reward behaviours similar to those seen in bvFTD including increased impulsivity, binge eating and alcohol or recreational drug abuse.

There are some limitations to this study. Owing to the small dimensions of the nucleus and the resolution of the MRI, it was not possible to distinguish between the lateral and medial habenula, and specifically locate the involvement within the nucleus. The small sample size did not allow us to further differentiate among the different genetic mutations in FTD and their potential different impact. Moreover, we did not systematically collect information about behaviours linked to reward processing, preventing us from investigating any possible correlation with the clinical symptoms. Further studies in larger genetic and pathologically confirmed cohorts are required to confirm the role of the habenula in bvFTD, together with studies aimed at defining the functional and structural connections of the habenula within the reward network.

In summary, we found that in bvFTD the region with the most atrophy in comparison to controls was the habenula and that this region is uniquely affected in this disorder in comparison with an age-matched AD cohort. We suggest that the habenula is an under-recognised area of importance in bvFTD and may be a key region involved in the development of abnormal reward processing.

## Supplementary Material

Web supplement
