# Supplementary material for: The habenula: an under-recognised area of importance in frontotemporal dementia?
Source: J Neurol Neurosurg Psychiatry. 2015 Nov 13;87(8):910–2. doi: 10.1136/jnnp-2015-312067 (PMC4975822; doi:10.1136/jnnp-2015-312067)
Supplement: Web supplement [file jnnp-2015-312067-s1.pdf]

SUPPLEMENTARY DATA

Imaging methods

Volumetric T1-weighted MRI was performed in all 45 subjects on a 3T scanner (Tim Trio, Siemens) with the following parameters: TR=2200ms, TI=900ms, TE=2.9ms, flip angle=10°, acquisition matrix=256x256 and spatial resolution=1.1mm. Raw T1-weighted images were transformed into standard space by a rigid registration to the Montreal Neurological Institute (MNI305) template,[10-12] using NiftyReg, revision #418 (Centre for Medical Image Computing, UCL: http://cmic.cs.ucl.ac.uk/home/software/).

Segmentations of the habenula were then performed manually on coronal slices of a volumetric T1-weighted MRI using NiftyMIDAS (Centre for Medical Image Computing, UCL: http://cmic.cs.ucl.ac.uk/home/software/) using the protocol as described below. The intrarater intraclass correlation coefficient for this segmentation protocol was 0.938 (95% confidence intervals: 0.771-0.984) and 0.909 (0.677-0.977) for left and right habenula respectively, tested in a sample of ten cognitively-normal controls, scanned using the same MRI protocol as the study participants. Dice overlapping and Jaccard similarity coefficient indexes were 0.83 (standard deviation 0.06) and 0.71 (0.08) respectively. Volumes of the habenula were computed from the manual segmentations performed in NiftyMIDAS.

In order to obtain volumes for the rest of the brain we performed a cortical parcellation using a multiatlas segmentation propagation approach following the brainCOLOR protocol combining regions of interest to calculate grey matter volumes of the entire cortex, separated into the frontal, temporal, parietal, occipital, cingulate, and insula cortices.[13] We also performed a subcortical parcellation using the Neuromorphometrics protocol for the hippocampus, amygdala, caudate,

putamen, pallidum, nucleus accumbens, thalamus and brainstem and a parcellation of the cerebellum using the Diedrichsen cerebellar atlas producing a measure for the entire cerebellum by combining regions of interest.[13]

All brain volumes were corrected for total intracranial volume (TIV), which was calculated using the Statistical Parametric Mapping (SPM) 12 software, version 6225 (www.fil.ion.ucl.ac.uk/spm), running under Matlab R2012b (Math Works, Natick, MA, USA). The TIV corrected volume of a specific structure for each subject "i" was computed as follows: Structure $volume_{corrected(i)}$ = Structure $volume_{raw(i)}$*TIV_{mean} / $TIV_{(i)}$, where "Structure $volume_{raw(i)}$" is the raw value of the structure of the subject "i", "TIV_{mean}" is the average TIV of the study group, and "$TIV_{(i)}$" is the TIV of the subject "i".

Detailed segmentation protocol for the habenula on 3T MRI

1. Introduction

This protocol describes how to manually segment the human habenula on volumetric T1-weighted magnetic resonance images (MRIs), combining the criteria described by Savitz et al., 2011 [5] and by Lawson et al., 2013 [6] (Supplementary Figure). The habenula contains relatively dense white matter plexuses, so it can be distinguished from the adjacent gray matter nuclei by its contrast (i.e. hyper-intensity) on MRI.

2. Segmentation procedures

2.1 Image Orientation/Registration/Standard space

The segmentations are made on MRIs rigidly registered to Montreal Neurological Institute (MNI) standard space.

2.2 Direction of segmentation

Segmentation proceeds on contiguous coronal slices in the caudo-rostral direction. For a slice thickness of 1 mm, approximately 4-5 slices include the habenula (Supplementary Figure).

3. Segmentation landmarks

The segmentation includes the lateral and medial habenular nuclei, which could not be reliably distinguished from each other and thus are combined into a single habenular region.

3.1 Most caudal slice

The habenular segmentation begins on the caudal slice containing the posterior commissure (or the habenular commissure), in which the habenula is present as opposed to cerebrospinal fluid or the most rostral extent of the pineal gland. The habenula is clearly evident as a pyramidal-shaped structure which bulges into the third ventricle along the ventromedial aspect of the thalamus.

3.2 Most rostral slice

The habenular segmentation ends at the most rostral slice where the bright habenular tissue is not visible as protruding into the cerebrospinal fluid of the third ventricle, while it appears the dorsal tip of the stria medullaris (the white matter track which delimits the ventromedial aspect of the medial thalamus). In this slice, the habenula is not visible as delimited ventrally and medially from the thalamus by the stria medullaris of thalamus.

3.3 Ventral boundary

In the most caudal slices, the ventral boundary is defined by the dorsal edge of the white matter of the posterior commissure (or the habenular commissure). In the most rostral slices, the habenula borders with the paraventricular nucleus of the thalamus.

3.4 Dorso-lateral boundary

The dorsal and lateral borders are defined by the mediodorsal thalamic nucleus, limitans nucleus or pretectal area in most caudal slices, and by the white matter of the stria medullaris of the thalamus in the rostral slices.

3.5 Medial boundary

The cerebrospinal fluid of the third ventricle is the prominent landmark to define the medial boundary of the habenula.

Supplementary Figure. Example of habenular segmentation on the ICBM152 2009c Nonlinear Symmetric - 1x1x1mm template (McConnell Brain Imaging Centre, Montreal Neurological Institute, McGill University). All 1 mm slices are shown, in the caudo-rostral direction.

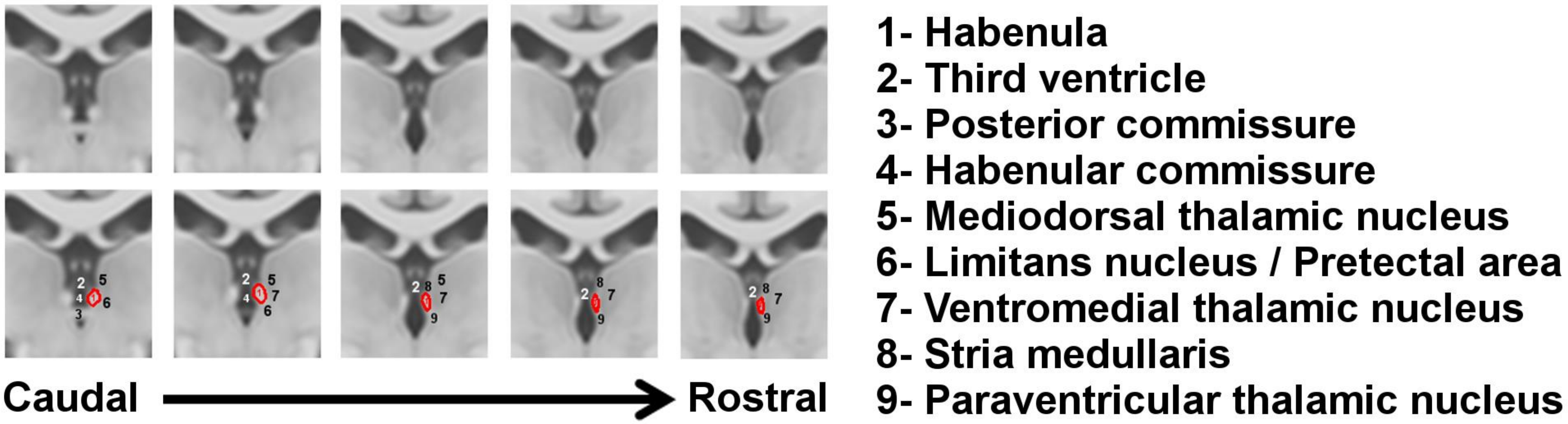

Supplementary Table. Volumetry of brain structures in 15 patients with bvFTD and 15 healthy control participants. Volumes are corrected for TIV. Values denote mean (standard deviation) volumes in mm^3. p-values denote significance on Mann-Whitney U test. *denotes significance after correction for multiple comparisons.

		**bvFTD (n=15)**	**Control (n=15)**	**p-value (*bvFTD vs Ctrl*)**	**% difference (*bvFTD vs Ctrl*)**
**Habenula**	**Right**	16 (3)	23 (2)	**<0.001***	30%
	**Left**	17 (2)	24 (2)	**<0.001***	28%
**Frontal Cortex**	**Right**	79850 (4699)	89306 (5454)	**<0.001***	11%
	**Left**	77130 (3817)	86667 (5570)	**<0.001***	11%
**Temporal Cortex**	**Right**	49270 (6741)	58717 (2766)	**<0.001***	16%
	**Left**	48265 (7676)	58919 (3158)	**<0.001***	18%
**Insula Cortex**	**Right**	5298 (684)	6918 (703)	**<0.001***	23%
	**Left**	5339 (791)	7263 (623)	**<0.001***	26%
**Cingulate Cortex**	**Right**	11270 (1138)	12405 (1123)	**0.029**	9%
	**Left**	12217 (872)	13727 (1273)	**<0.001***	11%
**Parietal Cortex**	**Right**	43906 (3592)	47309 (3513)	**0.019**	7%
	**Left**	44204 (2984)	47398 (4572)	**0.037**	7%
**Occipital Cortex**	**Right**	35453 (2235)	36857 (2988)	0.285	4%
	**Left**	33984 (1873)	35475 (2640)	0.074	4%
**Hippocampus**	**Right**	3645 (892)	4636 (486)	**0.001***	21%
	**Left**	3361(784)	4397 (337)	**<0.001***	24%
**Amygdala**	**Right**	731 (237)	971 (108)	**0.003***	25%
	**Left**	708 (206)	1015 (119)	**<0.001***	30%
**Caudate**	**Right**	3231 (620)	3526 (370)	**0.037**	8%
	**Left**	2944 (568)	3341(404)	**0.011**	12%
**Putamen**	**Right**	4290 (620)	4857 (560)	**0.004**	12%
	**Left**	4268 (579)	4872 (613)	**0.008**	12%
**Pallidum**	**Right**	803 (119)	850 (72)	0.267	5%
	**Left**	814 (118)	883 (98)	0.089	8%
**Nucleus accumbens**	**Right**	517 (103)	693 (65)	**<0.001***	25%
	**Left**	531 (131)	757 (77)	**<0.001***	30%
**Thalamus**	**Right**	5457 (675)	6351 (471)	**<0.001***	14%
	**Left**	5256 (646)	6206 (419)	**<0.001***	15%
**Brain Stem**		9109 (911)	9942 (647)	**0.009**	8%
**Cerebellum**		100761 (10296)	107356 (7454)	0.074	6%

Additional references for Supplementary Data

10. Evans AC, Collins DL, Milner B. An MRI-based stereotaxic atlas from 250 young normal subjects. Proc 22nd Annual Symposium, Society for Neuroscience. 1992; 18:408.

11. Evans AC., Marrett S, Neelin P, Collins L, Worsley K, Dai W. et al. Anatomical mapping of functional activation in stereotactic coordinate space. NeuroImage. 1992; 1:43-63.

12. Evans AC, Collins DL, Mills SR, Brown ED, Kelly RL, Peters TM. 3D statistical neuroanatomical models from 305 MRI volumes. Proc IEEE Nuclear Science Symposium and Medical Imaging Conference. 1993;1813-1817.

13. Cardoso MJ, Modat M, Wolz R, Melbourne A, Cash D, Rueckert D, Ourselin S. Geodesic information flows: spatially-variant graphs and their application to segmentation and fusion. IEEE TMI 2015, doi: 10.1109/TMI.2015.2418298.
